# Supplementary material for: Adaptive integrated intervention approaches for schistosomiasis elimination in Pemba: A 4-year intervention study and focus on hotspots
Source: PLoS Negl Trop Dis. 2025 Jun 2;19(6):e0013079. doi: 10.1371/journal.pntd.0013079 (PMC12129218; doi:10.1371/journal.pntd.0013079)
Supplement: S1 Text — (PDF) [file pntd.0013079.s001.pdf]

Microhematuria prevalence in hotspot implementation units

In 2021, 4.6% (31/669) of the children participating in the school-based survey in the five IUs considered hotspots tested positive for microhematuria (S1A Fig). Of the 669 children participating, nine (1.4%) children were diagnosed with large microhematuria. After one year of multidisciplinary interventions in these IUs, 6.8% (53/776) of the participating children tested microhematuria-positive, with 1.6% (12/776) having large microhematuria. In the newly classified four hotspot IUs in 2022, 3.5% (15/425) of the participating children in the school-based survey tested positive for microhematuria, with 0.7% (3/425) having large microhematuria. After one year of multidisciplinary interventions in the four hotspot IUs, the prevalence changed to 6.4% (28/441) and 0.2% (1/441) of children had large microhematuria. In the three newly classified hotspot IUs in 2022, 7.6% (30/395) of the children in the school-based survey tested positive for microhematuria, with 0.5% (2/395) large microhematuria. In the same schools, 2.0% (9/457) of the children in the school-based survey tested positive for microhematuria in 2024, with 0.4% (2/457) having large microhematuria.

In 2021, 6.9% (63/916) of the individuals participating in the household-based survey in the five IUs considered hotspots tested positive for microhematuria (S1B Fig). Of the 916 individuals participating, 2.0% (18/916) were diagnosed with large microhematuria. After one year of multidisciplinary interventions in these IUs, 8.6% (89/1044) of the participating individuals tested positive for microhematuria with 1.4% (14/1044) showing large microhematuria.

In the newly classified four hotspot IUs in 2022, 15.3% (121/800) of the participating individuals in the household-based survey tested positive for microhematuria, with 1.6% (13/800) having large microhematuria. After one year of multidisciplinary interventions in the

four hotspot IUs, the prevalence of microhematuria and of large microhematuria changed to 7.5% (66/879) and 1.6% (14/879), respectively.

In the three newly classified hotspot IUs in 2023, 10.9% (65/599) of the individuals participating in the household-based survey tested positive for microhematuria, with 1.5% (9/599) of the individuals having large microhematuria. In the same IUs, 17.1% (106/619) of the individuals tested positive for microhematuria in 2024, with 4.4% (27/619) of the individuals having large microhematuria.
