# Supplementary figures and images for: Adaptive integrated intervention approaches for schistosomiasis elimination in Pemba: A 4-year intervention study and focus on hotspots
Source: PLoS Negl Trop Dis. 2025 Jun 2;19(6):e0013079. doi: 10.1371/journal.pntd.0013079 (PMC12129218; doi:10.1371/journal.pntd.0013079)

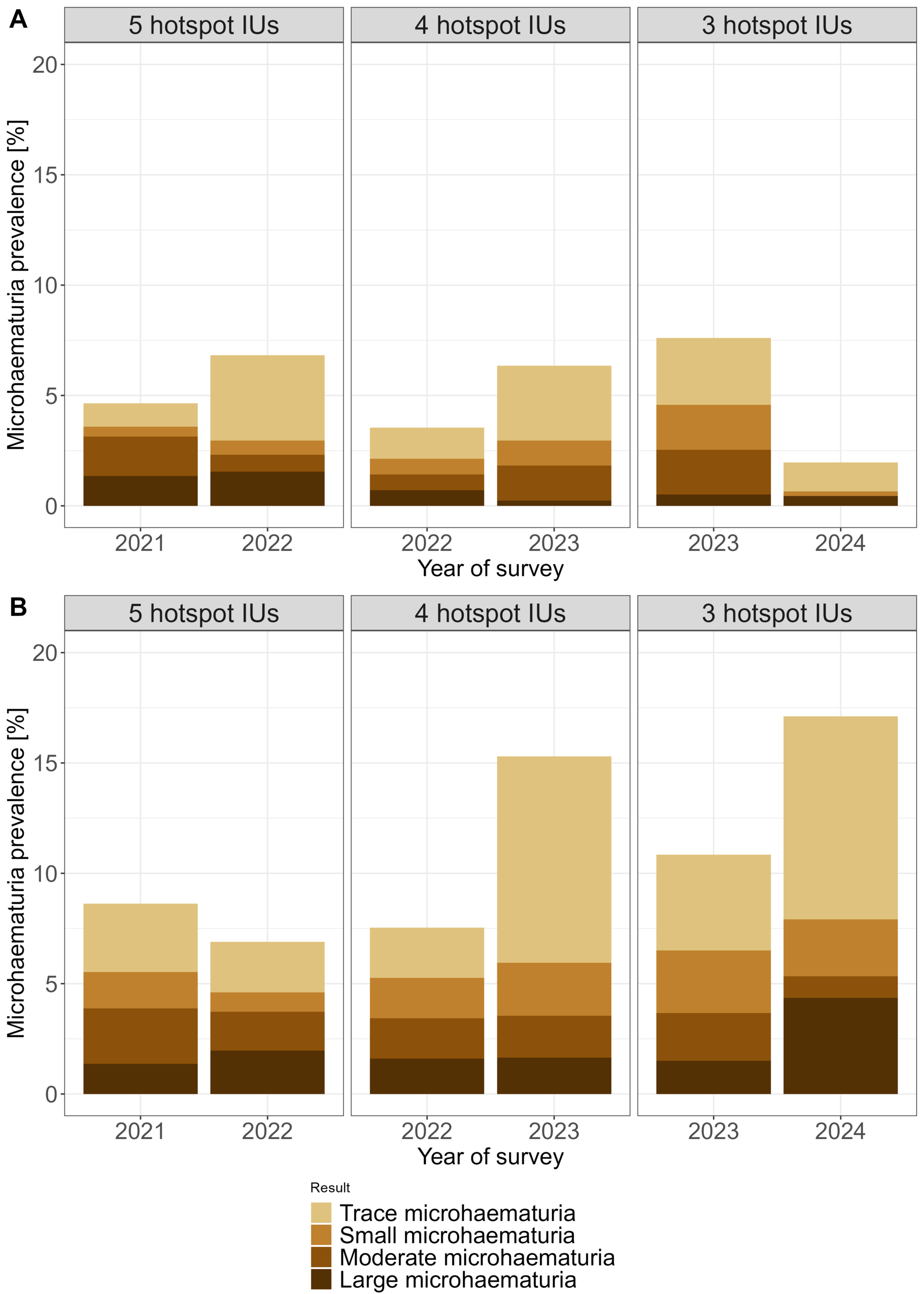

Supplement: S1 Fig — (TIF) [file pntd.0013079.s002.tif]
