# Supplementary material for: Adaptive integrated intervention approaches for schistosomiasis elimination in Pemba: A 4-year intervention study and focus on hotspots
Source: PLoS Negl Trop Dis. 2025 Jun 2;19(6):e0013079. doi: 10.1371/journal.pntd.0013079 (PMC12129218; doi:10.1371/journal.pntd.0013079)
Supplement: S2 STROBE Checklist — (PDF) [file pntd.0013079.s003.pdf]

STROBE Statement—checklist of items that should be included in reports of observational studies

	Item No.	Recommendation	Page No.	Relevant text from manuscript
**Title and abstract**	1	(*a*) Indicate the study's design with a commonly used term in the title or the abstract	1	Title: Adaptive integrated intervention approaches for schistosomiasis elimination in Pemba: a 4-year intervention study and focus on hotspots
		(*b*) Provide in the abstract an informative and balanced summary of what was done and what was found	1&2	**Methodology:** From 2020 to 2024, annual cross-sectional surveys were conducted in schools and communities in 20 implementation units (IUs), to assess the *S. haematobium* prevalence and monitor the impact of interventions. Based on the prevalence, the IUs were annually re-stratified into hotspot and low-prevalence IUs. In hotspots, mass drug administration in schools and communities, snail control and behavior change measures were implemented. Low-prevalence areas received surveillance-response interventions. With a random effects model, the association between *S. haematobium* infections and environmental and economic factors were assessed. Using risk layers based on the random effects model, hotspot areas were determined geographically.<br><br>**Principal Findings:** The overall *S. haematobium* prevalence in the 20 IUs changed from 1.2% (26/2200) in 2021 to 1.0% (27/2752) in 2024 in schools, and from 0.8% (31/3885) in 2021 to 1.2% (43/3711) in 2024 in communities. Across the study period, 8 IUs were considered a hotspot. The number of hotspot IUs decreased from 5 in 2021, to 4 in 2022, to 3 in 2023 but increased again to 5 in 2024. Some of the hotspot IUs resurged once interventions were adapted to surveillance-response. *S. haematobium* infections were significantly associated with residing <200m to a water body with *Bulinus* (Odds Ratio (OR): 14.1; 95% Confidence Interval (CI): 2.7-72.5), a very low economic score (OR: 4.3; 95% CI: 2.1-8.5) and living >2000m away from a road (OR: 2.3; 95% CI: 1.0-5.1).
**Introduction**				
Background/rationale	2	Explain the scientific background and rationale for the investigation being reported	6&7	However, the focality of *Schistosoma* transmission and a pronounced spatial heterogeneity in prevalence levels are a challenge for elimination [8, 12, 13]. Several countries that are approaching elimination contain areas where prevalences are low and other areas where transmission and infection remain high, despite repeated interventions [13-15]. For a progress towards elimination by an adequate use of resources, it will be of utmost importance to consider the local micro-epidemiology and to target and adapt interventions accordingly [16-18]. While in low-prevalence areas, MDA may be stopped and replaced by test-and-treat or other individualized interventions, remaining hotspot areas constitute a particular challenge for reaching the elimination goals and hence require comprehensive intervention strategies.
Objectives	3	State specific objectives, including any prespecified hypotheses	7	In the SchistoBreak study implemented from 2020 to 2024, we aimed to assess the contribution of three years of multidisciplinary intervention approaches that were targeted and adapted to the local micro-epidemiology, to urogenital schistosomiasis elimination in Pemba [19]. Here, we focused on the impact of all applied interventions on the overall prevalence in the study area and particularly on the impact of integrated MDA, snail control and behavior change interventions on the prevalence in

				hotspot areas. In addition, we identified factors that can help to explain the occurrence and persistence of hotspots.
**Methods**				
Study design	4	Present key elements of study design early in the paper	9	The SchistoBreak study was designed as a 4-year intervention study, with annual parasitological surveys and interjacent intervention periods [19]. The four annual parasitological surveys employed a cross-sectional sampling approach in schools and communities. The multidisciplinary interventions were targeted to the local micro-epidemiology of *S. haematobium*, as described below.
Setting	5	Describe the setting, locations, and relevant dates, including periods of recruitment, exposure, follow-up, and data collection	8	The SchistoBreak study was conducted on Pemba, an island in the Zanzibar archipelago of the United Republic of Tanzania, from 2020 to 2024. Pemba is divided into four districts, which contain 129 small administrative areas, called shehias [20]. The SchistoBreak study was conducted in the northern districts Wete and Micheweni, in a total of 20 shehias [19]. Each shehia in the study area was considered an IU. Based on the most recent Tanzanian population census, these 20 IUs had an estimated population size of approximately 95,000 people in 2022 [20]. In and around the SchistoBreak study area, 23 health facilities (21 primary health care units (PHCUs) and two hospitals) are located, providing basic and specialized healthcare services.
Participants	6	(*a*) *Cohort study*—Give the eligibility criteria, and the sources and methods of selection of participants. Describe methods of follow-up<br>*Case-control study*—Give the eligibility criteria, and the sources and methods of case ascertainment and control selection. Give the rationale for the choice of cases and controls<br>*Cross-sectional study*—Give the eligibility criteria, and the sources and methods of selection of participants	9	Eligible to participate in the surveys and interventions were all individuals who i) attended a school in the study area or lived in the study area, ii) were aged ≥4 years, and iii) provided a signed informed consent form for participation.
		(*b*) *Cohort study*—For matched studies, give matching criteria and number of exposed and unexposed<br>*Case-control study*—For matched studies, give matching criteria and the number of controls per case		
Variables	7	Clearly define all outcomes, exposures, predictors, potential confounders, and effect modifiers.	17 & 18	To assess the household-economic status of individuals participating in the household-based surveys, a principal component analysis (PCA) was conducted based on 13 variables indicating wealth (type of floor, type of toilet, type of cooking material, type of roof, type of drinking water, electricity

		Give diagnostic criteria, if applicable		availability, radio availability, refrigerator availability, television availability, mobile phone availability, bicycle availability, motorbike availability, car availability). To ensure the quality of the PCA, individuals with missing values in at least one of the 13 variables were excluded. The 13 variables were standardized to a mean of zero and a standard deviation of 1 to ensure that each variable contributed equally to the PCA. The PCA was conducted on the selected variables and the contribution of each variable to the principal components was evaluated by printing the loadings.<br><br>To determine the chance of being infected with *S. haematobium*, a random effects model was conducted with *S. haematobium* infection in the household-based survey as the dependent variable. The independent variables included demographic information (sex and age) and the first principal component indicating the household economic status, which was grouped through natural breaks. Further independent variables were the density of water bodies with *Bulinus* presence, standardized to a mean of zero and a standard deviation of 1, and the distance from the household to the next road as a proxy for the rural/urban location of the household. The IU was included in the model as the random effect. Odds ratios (ORs) with 95% confidence intervals (CIs) with a statistical significance at the 5% level were assessed.
Data sources/ measurement	8*	For each variable of interest, give sources of data and details of methods of assessment (measurement). Describe comparability of assessment methods if there is more than one group	16	For the examination of *S. haematobium* infections with the urine filtration method, all urine samples collected in parasitological surveys and surveillance activities were brought to the laboratory at PHL-IdC. Here, 10 ml of each urine sample were filtered through a 13 mm fabric filter (Sefar Ltd., Bury, United Kingdom), held by a Swinnex plastic filter holder (Millipore, Merck KGaA, Darmstadt, Germany), using a 10 ml plastic syringe. Subsequently, the filter was removed from the holder and examined under a light microscope to determine the presence and quantity of *S. haematobium* eggs. Samples containing 1 to 49 eggs per 10 ml of urine were classified as light-intensity infection, while those containing 50 or more eggs per 10 ml of urine were classified as heavy-intensity infection [37].
Bias	9	Describe any efforts to address potential sources of bias		NA
Study size	10	Explain how the study size was arrived at	9-11	Sample size calculations are described in the published study protocol [19].<br><br>**Cross-sectional parasitological school-based surveys**<br><br>For the school-based survey, the largest public primary school was selected in each IU. At baseline, 16 of the 20 IUs had a public primary school. In subsequent study years, 18 of the 20 IUs had a public primary school. In IUs without a public primary school, no school-based survey was conducted.<br><br>The school-based surveys were conducted in seven grades (nursery, grades 1-6) at each school. For each grade, a class was selected randomly for participation, based on a computer-generated list. For each grade, it was aimed to include 20 children. Accounting for a 20% dropout rate, 25 children were randomly selected per class. For randomization, children were asked to line up, sorted by sex. Subsequently, every third child from each line was selected for a total of 25 children. The study was explained to the children in Kiswahili, and they were given an information sheet along with informed

consent and assent forms. Demographic data, such as age and sex, were collected by the study team. On the following day, after submitting the signed informed assent and consent forms, the children were asked to provide a urine sample between 10 am and 2 pm. The urine samples were stored in a cool box and transported to the PHL-IdC.

Cross-sectional parasitological household-based surveys

A household-based cross-sectional survey was conducted in all 20 IUs in each study year. Aiming for 250 participants per IU, 70 housing structures per IU were randomly pre-selected in 2021, accounting for an average of five household members and a 30% drop-out rate [28, 29]. For the years 2022 to 2024, 80 housing structures were pre-selected accounting for a 40% drop-out rate based on experiences from the baseline survey [29]. For the selection of the housing structures, centroids of spatial polygons of buildings in Pemba were extracted from shape files, provided by the Zanzibar Commission for Lands to the Zanzibar Neglected Tropical Diseases Program, using ESRI ArcMap 10.6.1 [28]. Subsequently, the centroids to be included into the survey were randomly selected using R (www.r-project.org). With the combination of a mobile application for data collection (Open Data Kit (ODK, www.opendatakit.org) and a mobile application for offline navigation (Maps.Me, www.de.maps.me, in 2021 & 2022 and OrganicMaps, www.organicmaps.app, in 2023 and 2024), both installed on Samsung Galaxy A Tabs, the centroids of the housing structures were locatable by the study team in the communities.

Each IU was visited by the study team for three consecutive days. On day one, the housing structures were located. It was assessed whether the housing structures were residential houses or other housing structures, such as shops, mosques, and schools. In residential houses, the field enumerators explained the purpose of the study to present household members in Kiswahili. All eligible household members were invited to participate in the survey by signing the informed consent and assent forms and providing a fresh urine sample. A questionnaire to collect demographic data of all household members and additional information was conducted with one present adult household member. In 2022 to 2024, household-economic data were collected, such as the type of roof and floor, and possessions, such as a TV, radio, bicycle, or access to electricity at home. If some household members were not present during the first visit, urine cups were left with a present household member, marked with unique participant identifier codes and stickers with drawings to identify the intended recipient. The present household members were instructed to ask the returning household members to provide a urine sample and signed informed consent and assent forms the following morning.

On the second and third day, the study team revisited all households where they had previously distributed urine cups and consent forms to collect any remaining items.

Continued on next page

Quantitative variables	11	Explain how quantitative variables were handled in the analyses. If applicable, describe which groupings were chosen and why	18	To determine the chance of being infected with *S. haematobium*, a random effects model was conducted with *S. haematobium* infection in the household-based survey as the dependent variable. The independent variables included demographic information (sex and age) and the first principal component indicating the household economic status, which was grouped through natural breaks. Further independent variables were the density of water bodies with *Bulinus* presence, standardized to a mean of zero and a standard deviation of 1, and the distance from the household to the next road as a proxy for the rural/urban location of the household. The IU was included in the model as the random effect. Odds ratios (ORs) with 95% confidence intervals (CIs) with a statistical significance at the 5% level were assessed.
Statistical methods	12	(*a*) Describe all statistical methods, including those used to control for confounding	17 - 19	To assess the household-economic status of individuals participating in the household-based surveys, a principal component analysis (PCA) was conducted based on 13 variables indicating wealth (type of floor, type of toilet, type of cooking material, type of roof, type of drinking water, electricity availability, radio availability, refrigerator availability, television availability, mobile phone availability, bicycle availability, motorbike availability, car availability). To ensure the quality of the PCA, individuals with missing values in at least one of the 13 variables were excluded. The 13 variables were standardized to a mean of zero and a standard deviation of 1 to ensure that each variable contributed equally to the PCA. The PCA was conducted on the selected variables and the contribution of each variable to the principal components was evaluated by printing the loadings.<br><br>To determine the chance of being infected with *S. haematobium*, a random effects model was conducted with *S. haematobium* infection in the household-based survey as the dependent variable. The independent variables included demographic information (sex and age) and the first principal component indicating the household economic status, which was grouped through natural breaks. Further independent variables were the density of water bodies with *Bulinus* presence, standardized to a mean of zero and a standard deviation of 1, and the distance from the household to the next road as a proxy for the rural/urban location of the household. The IU was included in the model as the random effect. Odds ratios (ORs) with 95% confidence intervals (CIs) with a statistical significance at the 5% level were assessed. Since no economic data were collected in the household-based survey 2021, only complete data from 2022, 2023, and 2024 were included.
		(*b*) Describe any methods used to examine subgroups and interactions		
		(*c*) Explain how missing data were addressed	18	Since no economic data were collected in the household-based survey 2021, only complete data from 2022, 2023, and 2024 were included.
		(*d*) *Cohort study*—If applicable, explain how loss to follow-up was addressed<br>*Case-control study*—If applicable, explain how matching of cases and controls was addressed<br>*Cross-sectional study*—If applicable, describe analytical methods taking account of sampling strategy		

		(*e*) Describe any sensitivity analyses		
**Results**				
Participants	13*	(a) Report numbers of individuals at each stage of study—eg numbers potentially eligible, examined for eligibility, confirmed eligible, included in the study, completing follow-up, and analysed	19 & 20	In the four school-based cross-sectional surveys from 2021 to 2024, a total of 11614 children from 18 schools (16 in 2021) were randomly selected (Fig. 1). Of the 11614 registered children, two (0.02%) children were excluded from the analysis due to non-eligibility (<4 years old), 1385 (12.0%) children were excluded due to their absence on the day of urine collection and 56 (0.5%) were excluded due to refusal or not submitting a signed consent form. Finally, urine samples from 10171 children were collected and included in the parasitological analysis.<br><br>In the household-based surveys from 2021 to 2024, a total of 6200 housing structures were randomly selected in the 20 IUs (Fig. 1). Of those, 1464 (23.6%) were no households, in 701 (11.3%) households, nobody was at home at the time of visit, in 229 (3.7%) households, all individuals refused to participate, in 42 (0.7%) households, no adult person was at home at the time of visit and 22 (0.4%) housing structures were inaccessible, were not found or not visited. Hence, in total, 3742 residential houses and 18860 individuals were surveyed. Of the 18860 individuals, 5 (0.03%) were excluded due to non-eligibility (<4 years old), 3326 (17.6%) were excluded due to absence on the day of urine collection or since they did not submit a urine sample, and 58 (0.3%) individuals refused to participate or did not submit an informed consent form. Finally, urine samples from 15471 individuals were collected and included in the parasitological analyses.
		(b) Give reasons for non-participation at each stage	19	See above
		(c) Consider use of a flow diagram	20	Figure 1
Descriptive data	14*	(a) Give characteristics of study participants (eg demographic, clinical, social) and information on exposures and potential confounders	20 - 22	Of the 10171 children participating in the school-based surveys that were included in the analysis, 51.0% (5183/10171) were female, and 49.0% (4988/10171) were male (Table 1). The median age of the participating children was 9 (4-17) years. Of the 15471 individuals participating in the household-based survey included in the analysis, 55.0% (8503/15471) were female, and 45.0% (6968/15471) were male. The median age of the participating individuals was 17 (4-102) years.<br><br>Table 1
		(b) Indicate number of participants with missing data for each variable of interest		NA
		(c) *Cohort study*—Summarise follow-up time (eg, average and total amount)		NA
Outcome data	15*	*Cohort study*—Report numbers of outcome events or summary measures over time		NA
		Case-control study—Report numbers in each exposure category, or summary measures of exposure		NA

		Cross-sectional study—Report numbers of outcome events or summary measures	*23 & 24*	The results of the baseline school-based survey conducted in 2021 showed that 1.2% (26/2200) of the participating children were egg-positive for *S. haematobium*, with 0.2% (4/2200) having a heavy-intensity infection (Fig 2A). After one year of interventions in hotspot and low-prevalence areas, the overall prevalence changed to 0.9% (22/2527) in 2022, with 0.1% (3/2527) heavy-intensity infections. In 2023, 1.0% (27/2684) and 0.3% (9/2684) of the children were infected with *S. haematobium* and had heavy-intensity infections, respectively. In the final survey in 2024, 1.0% (27/2752) of the children tested egg-positive for *S. haematobium*, with 0.1% (3/2752) having a heavy-intensity infection.<br><br>The baseline household-based survey conducted in 2021 revealed a *S. haematobium* prevalence of 0.8% (31/3885) (Fig 2B). Among the individuals tested, 0.1% (3/3885) had a heavy-intensity infection. After one year of interventions, in 2022, 0.9% (34/3963) of the participants tested egg-positive, with 0.2% (7/3963) heavy-intensity infections. In 2023, the prevalence changed to 1.0% (40/3844), with 0.1% (5/3844) heavy-intensity infections. In the final year of the SchistoBreak study, 1.2% (43/3711) of the participants were egg-positive, and 0.1% (5/3711) had a heavy-intensity infection.<br><br>The results of the baseline school-based survey conducted in 2021 showed that 3.5% (78/2202) of the participating were microhaematuria-positive, with 0.7% (15/2202) having large microhaematuria (Fig 2A). After one year of interventions in hotspot and low-prevalence areas in 2022, the overall microhaematuria prevalence changed to 6.4% (163/2529), with 0.9% (23/2529) of children having large microhaematuria. In 2023, 5.4% (145/2680) of the children tested microhaematuria-positive, with 0.6% (17/2680) large microhaematuria. In the final survey of the SchistoBreak study conducted in 2024, 2.7% (73/2752) of the children were microhaematuria-positive, with 0.4% (11/2752) having large microhaematuria.<br><br>The baseline household-based survey conducted in 2021 revealed a microhaematuria prevalence of 5.8% (225/3885) among the participants (Fig 2B). Out of the 3885 individuals tested, 1.3% (49/3885) had large microhaematuria. After one year of interventions, in 2022, the microhaematuria prevalence was 12.0% (472/3963) with 1.2% (47/3963) large microhaematuria. In 2023, 9.4% (359/3844) of the participants tested microhaematuria-positive, with 1.1% (44/3844) large microhaematuria. In the final year of the SchistoBreak study, the microhaematuria prevalence was 10.9% (404/3711) with 2.2% (83/3711) large microhaematuria.<br><br>Figure 2 A&B
Main results	16	(*a*) Give unadjusted estimates and, if applicable, confounder-adjusted estimates and their precision (eg, 95% confidence interval). Make clear which confounders were adjusted for and why they were included	33 & 34	The standardized kernel density of water bodies with *Bulinus* presence was significantly associated with *S. haematobium* infections (OR: 1.8, 95% CI: 1.4-2.3), indicating that for each one standard deviation increase in the density, the odds of infection increased 1.8 times.<br>The odds of a *S. haematobium* infection were significantly higher for individuals with a low (OR: 2.5; 95% CI: 1.0-6.3), and very low economic score (OR: 4.1; 95% CI: 1.7-9.9), in comparison with individuals living in a household with a very high economic score.<br>A long distance from the house to a road was also a significant risk factor for a *S. haematobium* infection. Individuals living 500 m – 1 km away from the road (OR: 2.9; 95% CI: 1.5-5.6) and

		individuals living 1 – 2 km away from the road (OR: 4.7; 95% CI: 2.1-10.6) had higher odds of infection, compared with individuals living <50m away from a road.
	(*b*) Report category boundaries when continuous variables were categorized	NA
	(*c*) If relevant, consider translating estimates of relative risk into absolute risk for a meaningful time period	NA

Continued on next page

Other analyses	17	Report other analyses done—eg analyses of subgroups and interactions, and sensitivity analyses		NA
**Discussion**				
Key results	18	Summarise key results with reference to study objectives	35 & 36	Our results show that the adaptive intervention approach maintained the very low overall *S. haematobium* prevalence of ~1% (range: 0.8%-1.2%) in the schools and communities of the study area. Since heavy-intensity infections across the study area remained below 0.3% throughout all surveys, also the elimination of schistosomiasis as a public health problem was sustained. Yet, microhematuria (without trace) was detected in ~2% of students and in 3-6% of community members each year, pointing to residual morbidity due to urogenital schistosomiasis or other causes [38]. However, the adaptive intervention approach did not result in the interruption of transmission within the 4 study years.
Limitations	19	Discuss limitations of the study, taking into account sources of potential bias or imprecision. Discuss both direction and magnitude of any potential bias	34-37	Included in discussion
Interpretation	20	Give a cautious overall interpretation of results considering objectives, limitations, multiplicity of analyses, results from similar studies, and other relevant evidence	37 & 38	Importantly, while continued MDA and additional interventions can help to reduce further and maintain a relatively low prevalence level in (former) hotspot areas when applied with high coverage as done in our study, the risk for transmission and infection and hence a rebound in prevalence will remain as long as poverty persists and people do not have easy access to improved water sources that allow the washing of clothes and dishes and showering at home, and to sanitary infrastructure including improved toilets at home and in proximity to natural open waterbodies. Hence, reducing poverty, investing in WASH infrastructure and elevating the socio-economic standard of people in areas endemic for schistosomiasis will remain vital to achieve the elimination goals set by WHO for 2030 and improve global health equity.
Generalisability	21	Discuss the generalisability (external validity) of the study results	37	Also a study from China showed a recrudescence of *Schistosoma* infections after large-scale administration of praziquantel was stopped [39] and mathematical models indicate that the prevalence in high-risk areas likely rebounds once interventions cease [40]. Hence, decisions to reduce or stop interventions need to be taken with caution. Ideally, such decisions should not only be based on current prevalence data and thresholds, but also considering historical and long-term data, local knowledge, and environmental and social factors. Moreover, mathematical models may be used to predict the occurrence of hotspot areas and help to assign adequate interventions [41]. Knowing the underlying factors why hotspots are hotspots would support the prediction of such areas.
**Other information**				
Funding	22	Give the source of funding and the role of the funders for the present study and, if applicable, for the original study on which the present article is based	39	Funding for the study has been obtained from the Swiss National Science Foundation (SNSF; Bern, Switzerland) via a PRIMA grant (PR00P3_179753 / 1) of Stefanie Knopp. The funders had no role in study design, data collection and analysis, decision to publish, or preparation of the manuscript.

*Give information separately for cases and controls in case-control studies and, if applicable, for exposed and unexposed groups in cohort and cross-sectional studies.

Note: An Explanation and Elaboration article discusses each checklist item and gives methodological background and published examples of transparent reporting. The STROBE checklist is best used in conjunction with this article (freely available on the Web sites of PLoS Medicine at http://www.plosmedicine.org/, Annals of Internal Medicine at http://www.annals.org/, and Epidemiology at http://www.epidem.com/). Information on the STROBE Initiative is available at www.strobe-statement.org.
