# Supplementary material for: Adaptive integrated intervention approaches for schistosomiasis elimination in Pemba: A 4-year intervention study and focus on hotspots
Source: PLoS Negl Trop Dis. 2025 Jun 2;19(6):e0013079. doi: 10.1371/journal.pntd.0013079 (PMC12129218; doi:10.1371/journal.pntd.0013079)
Supplement: Attachment — Submitted filename: PNTD-D-24-01802R1_Point-by-point-response_FINAL_08.04.2025.pdf [file pntd.0013079.s008.pdf]

***Trippler et al.* "Adaptive integrated intervention approaches for schistosomiasis elimination in Pemba: a 4-year intervention study and focus on hotspots" (your reference no. PNTD-D-24-01802) – Point-by-Point response**

Allschwil, 8 April 2025

Dear Dr. Downs,

We were delighted to receive your e-mail dated 05 March 2025, which provided encouraging feedback from you and three external referees regarding the aforementioned manuscript. In the meantime, we carefully revised the manuscript in light of the reviewers' comments and suggestions and feel that the overall quality of our piece has been further improved.

Below, please find our point-by-point response, clearly indicating how and where in the manuscript changes have been made (line numbers). To assist you in readily tracking our amendments, we have highlighted all changes made in the revised version of our manuscript using a yellow marker. Additionally, we submit a clean version of the revised manuscript.

We very much hope that this revised version is now suitable for publication in *PLoS Neglected Tropical Diseases*. Hence, we look forward to your further disposition.

Yours sincerely,

Lydia Trippler and Stefanie Knopp

(on behalf of all authors)

****

Dear Dr. Knopp,

Thank you for submitting your manuscript to PLOS Neglected Tropical Diseases. After careful consideration, we feel that it has merit but does not fully meet PLOS Neglected Tropical Diseases's publication criteria as it currently stands. Therefore, we invite you to submit a revised version of the manuscript that addresses the points raised during the review process.

We thank you and the three external reviewers very much indeed for the thorough appraisal of our work and the overall positive feedback. We tried our level best to respond to each point and amended the manuscript accordingly. Please find our point-by-point response below.

Please submit your revised manuscript within 30 days May 04 2025 11:59PM. If you will need more time than this to complete your revisions, please reply to this message or contact the journal office at plosntds@plos.org. When you're ready to submit your revision, log on to https://www.editorialmanager.com/pntd/ and select the 'Submissions Needing Revision' folder to locate your manuscript file.

We have submitted our revised manuscript along with a point-by-point response to the reviewers' comments on April 8, 2025.

Please include the following items when submitting your revised manuscript:

* A rebuttal letter that responds to each point raised by the editor and reviewer(s). You should upload this letter as a separate file labeled 'Response to Reviewers'. This file does not need to include responses to any formatting updates and technical items listed in the 'Journal Requirements' section below.

We have uploaded a "Response to Reviewers" rebuttal letter, which responds to each point raised by the editor and reviewers to the *PloS Neglected Tropical Diseases* online system.

* A marked-up copy of your manuscript that highlights changes made to the original version. You should upload this as a separate file labeled 'Revised Manuscript with Track Changes'.

We have uploaded a marked-up copy of our manuscript that highlights changes made to the original version using track changes and labelled it "Revised Manuscript with Track Changes".

* An unmarked version of your revised paper without tracked changes. You should upload this as a separate file labeled 'Manuscript'.

We have uploaded an unmarked version of our revised paper without tracked changes and labelled it "Manuscript".

If you would like to make changes to your financial disclosure, competing interests statement, or data availability statement, please make these updates within the submission form at the time of resubmission. Guidelines for resubmitting your figure files are available below the reviewer comments at the end of this letter.

We did not change our financial disclosure, competing interests statement, or data availability statement.

We look forward to receiving your revised manuscript.

Kind regards,

Jennifer A. Downs, M.D., Ph.D.
Academic Editor
PLOS Neglected Tropical Diseases

Jong-Yil Chai
Section Editor
PLOS Neglected Tropical Diseases

Shaden Kamhawi
co-Editor-in-Chief
PLOS Neglected Tropical Diseases
orcid.org/0000-0003-4304-636XX

Paul Brindley
co-Editor-in-Chief
PLOS Neglected Tropical Diseases
orcid.org/0000-0003-1765-0002

Additional Editor Comments (if provided):

Please carefully review both the required and optional feedback from reviewers, which would strengthen the manuscript. Attention to the issue of the definition of hotspots, and strengths and limitations of using the definition that you used, is particularly important.

We have carefully reviewed both the required and optional feedback from all three reviewers and believe that the changes made have considerably improved the manuscript. We have paid particular attention to the issue of the definition of hotspots, which we responded to in the point-by-point response below and addressed in our revised manuscript in the discussion section (**see revised clean manuscript lines 653 - 667**).

Journal Requirements:

Please ensure that the CRediT author contributions listed for every co-author are completed accurately and in full.

At this stage, the following Authors/Authors require contributions: Lydia Trippler, Said Mohammed Ali, Mohammed Nassor Ali, Ulfat Amour Mohammed, Khamis Rashid Suleiman, Naomi Chi Ndum, Saleh Juma, Shaali Makame Ame, Fatma Kabole, Jan Hattendorf, and Stefanie Knopp. Please ensure that the full contributions of each author are acknowledged in the "Add/Edit/Remove Authors" section of our submission form.

The list of CRediT author contributions may be found here: https://journals.plos.org/plosntds/s/authorship#loc-author-contributions

The CRediT author contributions listed for every co-author of our manuscript are completed accurately and in full.

****

REVIEWER REPORTS

Reviewer #1: The paper is good and described the impact of multiple intervention implemented to control schistosomiasis in areas with low prevalences. The work is relevant and deserve to be published. Furthermore, the research assess how poverty can contribute to continual transmission of this disease. To reach eradication goals, poverty must be addressed.

We thank Reviewer #1 for highlighting the relevance of our work and for recommending its publication. We very much appreciate the thorough appraisal and detailed feedback on our manuscript. We addressed all the points and feel that the overall quality of our piece has been further improved.

Reviewer #2: This is a Research Article by Dr. Trippler and colleagues reporting on the SchistoBreak study performing a 4-year prospective adaptive intervention trial for Schistosoma haematobium elimination in low prevalence settings in Pemba, Tanzania. The authors applied a multi-disciplinary intervention (bi/annual MDA, snail control, behavioral change activities) to "hotspot" locations (defined as >3% school based prevalence or >2% household prevalence by urine microscopy) and surveillance / monitoring (with some directed test-and-treat) in non-hotspots based on annual repeated cross-sectional prevalence surveys across 20 locations. They found that while Schistosoma haematobium prevalence remained low with the multi-disciplinary intervention in hotspot locations, there was not elimination of transmission and prevalence remained overall the same. This is an important article reporting on a well conducted and impressive study on a pressing topic of elimination in the field of schistosomiasis. I would like to congratulate the authors and their team for completing this study. I hope my comments can be helpful and constructive.

We are very happy that Reviewer #2 considers our study as impressive and commends our work as an important and pressing topic in the field of schistosomiasis elimination. We thank Reviewer #2 for his/her very valuable feedback that helped to further improve the content of our manuscript. Our response to the comments is indicated below.

Major comments:

--The definition of “hotspot” is highly variable in the literature of Schistosoma and beyond and the authors definition is different from others (e.g., WHO). For their purposes, it makes sense. However, it would be helpful to clarify in the Methods the justification for their definition, and also explicitly state that urine microscopy was used for the prevalence measurement. Furthermore, some additional Discussion on alternative hotspots definitions could be helpful (i.e., most are often used in the context of “control” rather than “elimination”). My final point is that this binary classification of hotspot when the prevalence threshold is so low is probably prone to random variation (i.e., a few positive participants may change the classification); when considering imperfect diagnostic sensitivity, drug efficacy, etc, it would be helpful to have some comments from the authors on the robustness of this classification, so that some fluctuation in number of hotspots is not over-interpreted.

Reviewer#2 raises an important point, the definition of hotspots. We fully agree that the definition of "hotspots" varies across the literature and that our threshold differs from those used in other studies [1,2]. The WHO definition of “persistent hot spots”, which are areas with a baseline prevalence ≥ 50% in school-aged children (SAC), was only provided in 2022 [3], and hence several years after our study protocol for the SchistoBreak study was written and published [4]. For Zanzibar, which is an elimination setting where mass treatment programs with praziquantel started more than 20 years ago, the context is different, and this definition for baseline prevalences cannot be applied anymore.

In line with Reviewer#2’s suggestion, we have now provided a justification for our hotspot definition in the Method’s section as follows and explicitly state that urine microscopy was used for the prevalence measurement: “To target multidisciplinary intervention strategies for urogenital schistosomiasis elimination to the local micro-epidemiology of *S. haematobium* in Pemba, the 20 IUs were classified into hotspot and low-prevalence IUs. The selection of thresholds used to distinguish a “hotspot” IU from a low-prevalence IU in this study was based on age-dependent infection rates [4]. Every IU with a prevalence of ≥3% in the school-based survey and/or a prevalence of ≥2% in the household-based cross-sectional survey was classified as a hotspot IU. Every IU with a prevalence of <3% and <2% in the school-based and household-based survey, respectively, was classified as a low-prevalence IU. The prevalence estimates for the thresholds were based on the results of a single urine filtration. The classification of hotspot and low-prevalence IUs and respective intervention approaches was renewed annually in line with survey results. Of note, the thresholds were set at such a low prevalence levels since i) Pemba is an elimination setting with a very low overall prevalence [5,6] and ii) this study was the first study conducted in Pemba that stopped MDA in designated low-prevalence IUs to evaluate a surveillance-response approach including a test-treat-test-track-test-treat (5T) intervention [4,7]. We hence aimed to minimize the risk of recrudescence and respond rapidly to any increase in prevalence in IUs.” (**see revised clean manuscript, lines 210 – 225**).

Since we provided a clear justification for our thresholds in the method section of the revised manuscript and amended the discussion in light of the reviewers’ comments as indicated below,

we did not add any discussion that reflects on the differences in our thresholds versus other definitions of hotspots.

Yet, as suggested by Reviewer#2, we indicate in the discussion that " … one also has to note that the binary classification of hotspot and low-prevalence IUs as applied in our study is likely prone to random variation, particularly since our prevalence threshold was set very low and a few more or less positive participants may have caused a change in the classification. Moreover, when assigning interventions based on prevalence thresholds, it is important to be aware that the diagnostic methods used to determine the prevalence are imperfect and, when insensitive methods such as a single urine filtration are applied, the true prevalence is very likely underestimated. Consequently, there is a risk for ceasing interventions such as MDA too early, resulting in recrudescence." (**see revised clean manuscript, lines 666 – 673**).

--The study would benefit from additional discussion on other explanations for why this well-conducted and thought-out intervention did not eliminate transmission. For example, are participants traveling and getting infected elsewhere outside the implementation unit? Praziquantel efficacy is imperfect. Are we sure that all participants were treated in the prior MDA (given the design is a repeated cross-sectional and not the same cohort of individuals)? Did they all accept treatment? Animal reservoir? Size of the transmission network? We agree with the reviewer and added the following paragraph to the discussion: "Clearly, achieving elimination in a formerly highly endemic area such as Pemba is not easy, especially if resources are limited. Intervention approaches have to be selected and adapted very carefully to the local micro-epidemiology to use resources adequately and yet avoid recrudescence. If zero autochthonous infections in humans is the goal in Pemba or elsewhere, it will be important to apply comprehensive integrated intervention packages with a high coverage across the island. But even then, one has to be aware of factors that jeopardize elimination. Reasons for the persistence of transmission and recurrence of infections are manifold. In Pemba, the large number of streams and ponds and the presence of the intermediate host snail *Bulinus* in many of these waterbodies provide an ideal and continuous transmission environment for S. haematobium [8,9]. Snail control with niclosamide helped to reduce the number of *Bulinus* in our study area, but snails quickly returned and can thus contribute to maintaining transmission [10]. Animal reservoirs, such as cattle transmitting *S. bovis* and the potential for hybridization, should be considered as possible contributors to recrudescence [11,12]. Behavior change interventions as applied in our hotspot IUs successfully improved the knowledge, attitude and practices of exposed children, but need to be complemented by improved access to water and sanitation to contribute successfully to elimination [13,14]. The MDA coverage in the schools and communities may be high as in our study, but will not reach all infected individuals, either due to insufficient coverage, non-attendance, or non-compliance [15-18]. Moreover, due to an imperfect praziquantel efficacy [19], infected individuals who participate in the MDA may not be fully cured and continue to excrete viable eggs that can perpetuate transmission. Additionally, in our study, the surveillance-response approach that was applied in low-prevalence IUs had only a moderate sensitivity and several infected individuals may not have been treated [20]. Since people are mobile and may move within and between the Zanzibar

islands and abroad, transmission may be reintroduced in areas free of schistosomiasis." (**see revised clean manuscript, lines 691 – 715**).

Minor comments (most are just suggestions/optional):

--Abstract: In Principal findings, why not report 95% CI instead of the counts?

We thank Reviewer#2 for this suggestion and inserted the 95% CIs in the abstract. Of note, we kept the counts data since we feel it is important to highlight the large number of participants in our study.

--Some discussion on generalizability of these findings (Pemba island) to other settings would be helpful.

We thank Reviewer#2 for this suggestion. To highlight generalizability, we amended the conclusion of the abstract as follows: "To achieve complete elimination in Pemba and elsewhere, poverty needs to be reduced and investments in global health equity, including the water sanitation and hygiene infrastructure, are essential." **(see revised clean manuscript, lines 56 – 58).**

Moreover, in the discussion we have compared our results from Pemba with those from e.g. China (**see revised clean manuscript, lines 663 – 664**), Côte d'Ivoire, or Sudan (**see revised clean manuscript, lines 688 – 689**) and indicated that "Many of these factors are not specific for Pemba, but may consist a barrier to elimination also in other settings." **(see revised clean manuscript, lines 715 – 716).**

--Were there differences in timing of the relationship between MDA and the prevalence survey that could explain some variation in year to year results?

Our first cross-sectional survey was implemented from Nov 2020 to Feb 2021 and hence commenced 3 months after an MDA, which was implemented in August 2020. Following the study design, the following surveys were implemented annually at the same time of the year. The intervention periods, where also the MDAs were conducted, were implemented in the months between the surveys. While the MDAs were not always conducted in exactly the same months, we tried to keep the timing between MDA and subsequent surveys in schools and communities that had received MDA as equal as possible throughout the study period.

--Were any participants tested with CAA or PCR? If not, would add this as a limitation (e.g., the prevalence threshold is based on a diagnostic test with imperfect sensitivity).

We thank Reviewer#2 for highlighting the imperfect diagnostics. None of the participants of the cross-sectional surveys were tested with CAA and PCR. In light of the reviewer's suggestion, we amended the discussion as follows: "…when assigning interventions based on prevalence thresholds, it is important to be aware that the diagnostic methods used to determine

the prevalence are imperfect and, when insensitive methods such as a single urine filtration are applied, the true prevalence is very likely underestimated" (**see revised clean manuscript, lines 669 – 672**) and "Highly sensitive and specific tests should be applied to determine the prevalence on which the threshold and hence intervention implementation are based [21]." (**see revised clean manuscript, lines 676 – 677**).

--Data visualization: Could add color legend to Figure 3. I found Figure 4 hard to understand, and perhaps some more explanation in the legend could help.

We thank the reviewer for pointing out that Figure 3 and Figure 4 are hard to understand. To improve clarity, we revised as follows:

"Figure 3. Annual change in low-prevalence (orange) and hotspot (red) implementation units (IUs)." (**see revised clean manuscript, lines 487 – 488**).

"**Fig 4. *Schistosoma haematobium* prevalence in hotspot implementation units (IUs) in annual school-based surveys (A) and household-based surveys (B) of the SchistoBreak study in Pemba, Tanzania, from 2021 to 2024.** The figure shows the *S. haematobium* prevalence in hotspot IUs in the surveys before and after implementation of a comprehensive integrated intervention package. The number of hotspot IUs changed annually in line with the SchistoBreak study design." (**see revised clean manuscript, lines 544 – 549**).

--Is there any other cause of the microhematuria in this population?

We thank the reviewer for this question. Indeed, besides urogenital schistosomiasis there may be other causes of microhematuria in our study population. We have amended the discussion as follows: "Yet, microhematuria (without trace) was detected in ~2% of students and in 3-6% of community members each year, pointing to residual morbidity due to urogenital schistosomiasis or due to other causes, such as menstruation, urinary tract infections or bladder-stones [22,23]." (**see revised clean manuscript, lines 639 – 642**).

--Apologies if I missed this, but a figure plotting the resurgence of prevalence in nonhotspots given surveillance/monitoring would be quite helpful, as well as any unique features in those locations.

There is no need for Reviewer#2 to apologize. The resurgence of prevalence in low-prevalence IUs is indicated in Figure 3. However, there is no more detailed presentation since our focus in this paper is the hotspot IUs, and the results of the surveillance-response interventions in low-prevalence IUs will be presented in a manuscript that is currently in preparation.

--The authors looks at risk factors for individual infection, but what about risk factors for being a hotspot IU vs non-hotspot IU?

We have indeed looked at risk factors for individual infection. In addition, we also measured risk factors for an IU being a hotspot IU, as shown in Figure 7 (**see revised clean manuscript, lines 619 –629**), where we combined several risk factors that proved to be significant indicators for infection status, which aligns with areas being a hotspot IU.

Thank you for the opportunity to review this important work.

We thank Reviewer#2 for taking the time to review our manuscript and for providing highly valuable comments and suggestions that helped to improve our manuscript considerably.

Reviewer #3: The authors provide a valuable contribution to the dearth of evidence for "integrated management" that the WHO is driving. It is incredibly noteworthy that the "integrated" approach did nothing. There is so little evidence to back this drive from the WHO across so many diseases, and it is nice to see an attempt to provide some evidence towards this. We thank Reviewer#3 to acknowledge that our work provides a valuable contribution to the evidence base for integrated interventions against schistosomiasis. In line with a comment from Reviewer#2, we have indicated several explanations, why it is hard to achieve interruption of transmission, also with an integrated approach. The manuscript now contains the following information: "Clearly, achieving elimination in a formerly highly endemic area such as Pemba is not easy, especially if resources are limited. Intervention approaches have to be selected and adapted very carefully to the local micro-epidemiology to use resources adequately and yet avoid recrudescence. If zero autochthonous infections in humans is the goal in Pemba or elsewhere, it will be important to apply comprehensive integrated intervention packages with a high coverage across the island. But even then, one has to be aware of factors that jeopardize elimination. Reasons for the persistence of transmission and recurrence of infections are manifold. In Pemba, the large number of streams and ponds and the presence of the intermediate host snail *Bulinus* in many of these waterbodies provide an ideal and continuous transmission environment for *S. haematobium* [8,9]. Snail control with niclosamide helped to reduce the number of *Bulinus* in our study area, but snails quickly returned and can thus contribute to maintaining transmission [10]. Animal reservoirs, such as cattle transmitting S. bovis and the potential for hybridization, should be considered as possible contributors to recrudescence [11,12]. Behavior change interventions as applied in our hotspot IUs successfully improved the knowledge, attitude and practices of exposed children, but need to be complemented by improved access to water and sanitation to contribute successfully to elimination [13,14]. The MDA coverage in the schools and communities may be high as in our study, but will not reach all infected individuals, either due to insufficient coverage, non-attendance, or non-compliance [15-18]. Moreover, due to an imperfect praziquantel efficacy [19], infected individuals who participate in the MDA may not be fully cured and continue to excrete viable eggs that can perpetuate transmission. Additionally, in our study, the surveillance-response approach that was applied in low-prevalence IUs had only a moderate sensitivity and several infected individuals may not have been treated [20]. Since people are mobile and may move within and between the Zanzibar islands and abroad, transmission may

be reintroduced in areas free of schistosomiasis. Many of these factors are not specific for Pemba, but may consist a barrier to elimination also in other settings.

Hence, while continued MDA and additional interventions can help to reduce further and maintain a relatively low prevalence level in (former) hotspot areas, the risk for transmission and infection and hence a rebound in prevalence will remain as long as health literacy is low and poverty persists. Transmission and infection will continue as long as people do not understand the transmission routes and health consequences of schistosomiasis, and do not have easy access to infrastructure that allows the washing of clothes and dishes and showering with clean water at home, and to sanitary facilities including improved toilets at home and in proximity to natural open waterbodies. Reducing poverty, investing in WASH infrastructure and elevating the health literacy and socio-economic standard of people in areas endemic for schistosomiasis will remain vital to achieve the elimination goals set by WHO for 2030 and improve global health equity." (**see revised clean manuscript, lines 691 – 727**).

My major qualm with this paper is that I am not sure I agree with your use of the term hotspot. You can have a persistent transmission hotspot where prevalence is still in your "low prevalence" threshold…your groupings are almost operating on different units of observation…And in some way I think this is why you see your hotspots coming back and going away in a way that to me fundamentally just seems quite random. There is no difference between these groups, the whole dataset is a low but consistent force of infection with some noise.

We thank the reviewer for flagging his/her concerns. Also Reviewer#2 was concerned about the definition and classification of hotspots in our study, using prevalence thresholds at very low levels. In light with the reviewers comments, we have amended our discussion as follows: " … one also has to note that the binary classification of hotspot and low-prevalence IUs as applied in our study is likely prone to random variation, particularly since our prevalence threshold was set very low and a few more or less positive participants may have caused a change in the classification. Moreover, when assigning interventions based on prevalence thresholds, it is important to be aware that the diagnostic methods used to determine the prevalence are imperfect and, when insensitive methods such as a single urine filtration are applied, the true prevalence is very likely underestimated. Consequently, there is a risk for ceasing interventions such as MDA too early, resulting in recrudescence." **(see revised clean manuscript, lines 666 – 673).**

We do, however, not agree with the reviewer that there is no difference between our IU groups and that our whole dataset shows a low but consistent force of infection with some noise. In a previous study conducted in Zanzibar, certain areas consistently showed a rebound in prevalence shortly after interventions were stopped [5]. These increases in prevalences were not random fluctuations, but rather patterns observed repeatedly in the same locations over decades. Moreover, Figure 7 illustrates that there are indeed differences between areas in terms of transmission dynamics and infection risk, supporting the notion that some IUs face a higher force of infection than others. Moreover, these areas also exhibit environmental and sociodemographic differences, such as higher density of water bodies, and individuals with

lower socioeconomic status, which are described in the current manuscript **(see revised clean manuscript, lines 618 – 629).**

Of note, we appreciate the reviewer's thoughtful critique and hope that our clarification helps to better explain the rationale behind our classification and interpretation.

Figure 7 maps - it would help if these were zoomed out so that it is easier to see where your IUs fall in terms of the whole island.

We thank Reviewer#3 for this suggestion and have inserted an overview map into Figure 5, showing the location of our study sites were located in the north of Pemba **(see revised clean manuscript, Figure 5).**

Line numbers would make your feedback easier.

We apologize that we did not insert line numbers into the previous version of our manuscript. The revised version contains line numbers throughout all pages.

When you say you left out those with missing values for the PCA – how many did you start with and how many did you omit? Your sample sizes are not clear to me, I see you "aimed for 250 and 80 houses" but this isn't a clear number of participants…I see now it is in your results section, I would find this easier to process in the methods section so I can appraise your methods more efficiently.

It would be nice to see your PCA output so that we can see the clustering of PCA1.

We have now added information about the number of individuals included in the surveys from 2022 to 2024 (11576) and about how many of those had complete values in the manuscript (11518) and were therefore included in the PCA. The added information now reads as follows: "During the annual cross-sectional household-based surveys from 2022 to 2024, a total of 11576 individuals were included in the surveys, of whom 11518 community members were tested for *S. haematobium* infection using urine filtration." (**see revised clean manuscript, lines 553 – 555**).

Below, please find a boxplot figure showing the PC1 versus the infection status of the participants and a biplot of PC1 and PC2.

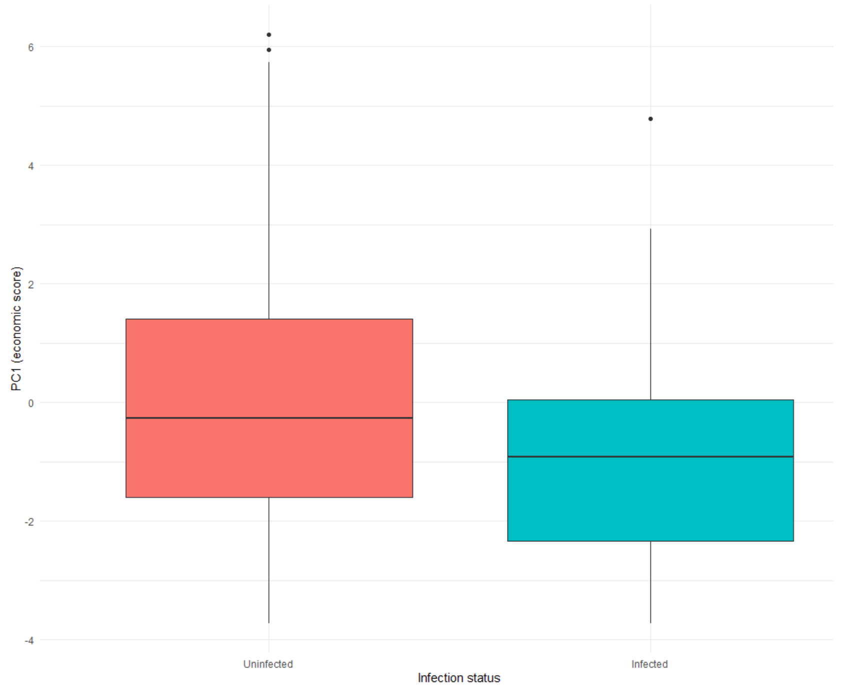

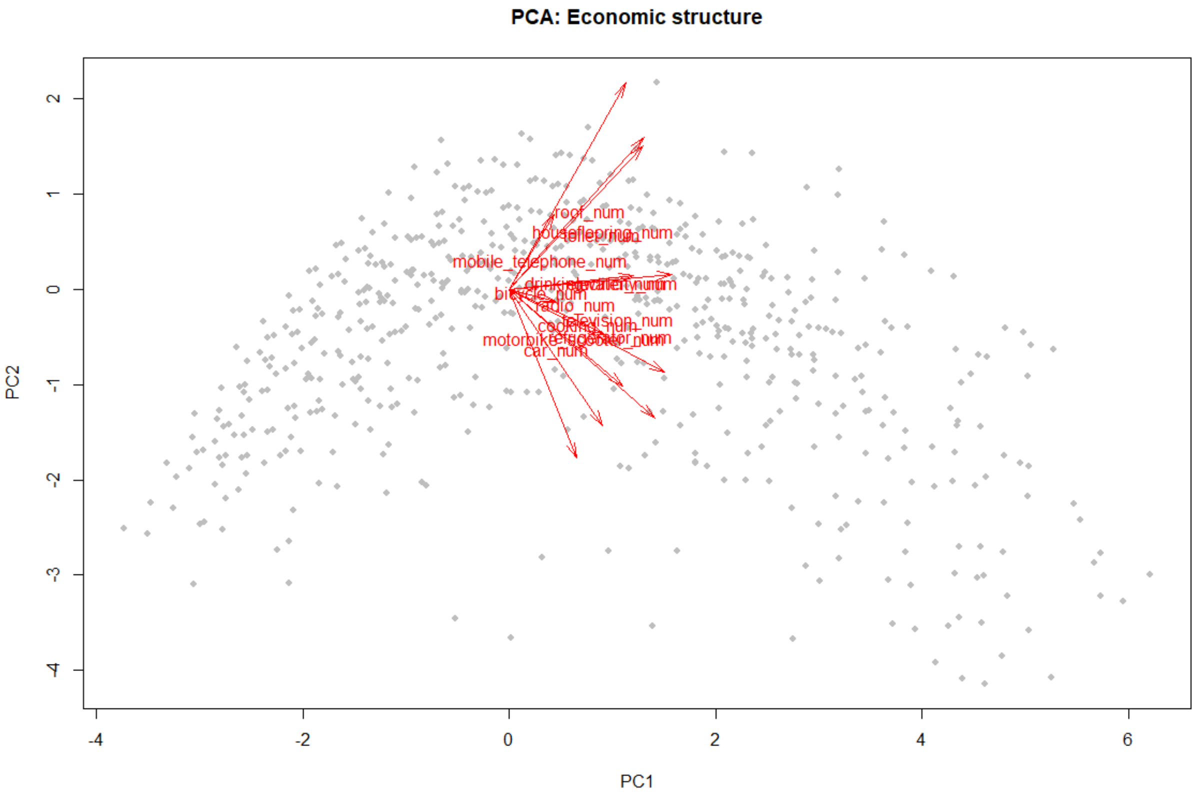

Indeed, we did not include the full sample size calculation in this manuscript since the sample size calculation is explained in our published study protocol, to which we refer as follows in the methods section: "Sample size calculations are described in the published study protocol [4]." **(see revised clean manuscript, lines 148 – 149).**

In the study protocol, the sample size calculations are described as follows: “We run a series of simulations to assess how precisely the sensitivity of the surveillance system can be estimated. We modelled different scenarios with varying prevalences in schoolchildren as well as in the general population, different values for clustering and different assumptions for diagnostic test accuracy and surveillance system performance. The results revealed that sampling an average of 140 schoolchildren and 250 community members in each of 15 low-prevalence shehias in the cross-sectional surveys enables us to estimate the prevalence with a median precision (defined as one half length of the confidence interval) of 10 to 15 percentage-points in schoolchildren and of 5 to 7 percentage-points in the general population. The bias (defined as difference between the sensitivity calculated using the apparent prevalence observed during cross-sectional surveys and the sensitivity calculated using the "true" apparent prevalence observed during the surveillance activities) had a median of zero and an interquartile range from -0.05 to 0.05. Hence, conducting the cross-sectional surveys in the schools and communities of all 20 study shehias, the yearly total sample is 7800 individuals (ca. 10% of the population, based on an average of 4000 inhabitants per shehia).” [4]. To keep the manuscript concise, we did not repeat this explanation in the revised version of our manuscript.

Figure resubmission:

While revising your submission, please upload your figure files to the Preflight Analysis and Conversion Engine (PACE) digital diagnostic tool, https://pacev2.apexcovantage.com/. PACE helps ensure that figures meet PLOS requirements. To use PACE, you must first register as a user. Registration is free. Then, login and navigate to the UPLOAD tab, where you will find detailed instructions on how to use the tool. If you encounter any issues or have any questions when using PACE, please email PLOS at figures@plos.org. Please note that Supporting Information files do not need this step. If there are other versions of figure files still present in your submission file inventory at resubmission, please replace them with the PACE-processed versions.

We have checked our figures with PACE and confirm that they meet PLOS requirements.

Reproducibility:

To enhance the reproducibility of your results, we recommend that authors of applicable studies deposit laboratory protocols in protocols.io, where a protocol can be assigned its own identifier (DOI) such that it can be cited independently in the future. Additionally, PLOS ONE offers an option to publish peer-reviewed clinical study protocols. Read more information on sharing protocols at https://plos.org/protocols?utm_medium=editorial-email&utm_source=authorletters&utm_campaign=protocols

Our study protocol is published in BMC Infectious Diseases (https://doi.org/10.1186/s12879-021-06620-8) [4].

Minor observations

- Can author present the data of MDA coverage and compliance to treatment

We thank the reviewer for this suggestion. Unfortunately, we do not have any reliable data on compliance. However, we present information on coverage as we received it from the NTD Program of the Zanzibar Ministry of Health **(see revised clean manuscript, Table 2)**.

- Line "8-10mg Niclosamide in 1l – write one litre (it looks like 11)

We thank the reviewer for this suggestion, and revised to "one liter" **(see revised clean manuscript, line 253).**

- Laboratory Examination:- give details of the classification of small and large

We have revised our explanation in the method section as follows: "The examination results were graded as microhematuria negative, or as trace, small (+), moderate (++), or large (+++) microhematuria, according to the manufacturer's color chart." **(see revised clean manuscript, lines 328 – 330).**

- Results:- page 23- line 18- what is large microhaematuria?

The term "large microhematuria" is provided by the manufacturer and equivalent to the highest possible grade (+++) of microhematuria that Hemastix can detect. We have revised the manuscript and included this explanation as indicated in our response to the previous comment.

- Line 108-118 – "with heavy intensity of infection

The definition is explained in our method section as follows: "Samples containing 1 to 49 eggs per 10 ml of urine were classified as light-intensity infection, while those containing 50 or more eggs per 10 ml of urine were classified as heavy-intensity infection [24]." **(see revised clean manuscript, lines 324 – 326).**

References

1. Pennance T, Person B, Muhsin MA, Khamis AN, Muhsin J, Khamis IS, et al. Urogenital schistosomiasis transmission on Unguja Island, Zanzibar: characterisation of persistent hot-spots. Parasit Vectors. 2016;9(1): 646.
2. Kittur N, Binder S, Campbell CH, King CH, Kinung'hi S, Olsen A, et al. Defining persistent hotspots: areas that fail to decrease meaningfully in prevalence after

multiple years of mass drug administration with praziquantel for control of schistosomiasis. Am J Trop Med Hyg. 2017;97(6): 1810-1817.
3. World Health Organization. WHO guideline on control and elimination of human schistosomiasis. In: Organization WH, editor.^editors. ed. Geneva: 2022. p. 142.
4. Trippler L, Hattendorf J, Ali SM, Ame SM, Juma S, Kabole F, et al. Novel tools and strategies for breaking schistosomiasis transmission: study protocol for an intervention study. BMC Infect Dis. 2021;21(1): 1024. Epub 2021/10/02.
5. Trippler L, Ame SM, Hattendorf J, Juma S, Abubakar S, Ali SM, et al. Impact of seven years of mass drug administration and recrudescence of *Schistosoma haematobium* infections after one year of treatment gap in Zanzibar: Repeated cross-sectional studies. PLoS Negl Trop Dis. 2021;15(2): e0009127. Epub 2021/02/13.
6. Knopp S, Ame SM, Person B, Hattendorf J, Rabone M, Juma S, et al. A 5-year intervention study on elimination of urogenital schistosomiasis in Zanzibar: Parasitological results of annual cross-sectional surveys. PLoS Negl Trop Dis. 2019;13(5).
7. Trippler L, Taylor L, Ali MN, Najim SO, Khamis KS, Hattendorf J, et al. Test-treat-track-test-treat (5T) approach for *Schistosoma haematobium* elimination on Pemba Island, Tanzania. BMC Infect Dis. 2024;24(1): 661. Epub 20240702.
8. Stothard IR, Rollinson D. Molecular characterization of *Bulinus globosus* and *B. nasutus* on Zanzibar, and an investigation of their roles in the epidemiology of *Schistosoma haematobium*. Trans R Soc Trop Med Hyg. 1997;91(3): 353-357.
9. Savioli L, Dixon H, Kisumku UM, Mott KE. Control of morbidity due to *Schistosoma haematobium* on Pemba island: programme organization and management. Trop Med Parasitol. 1989;40(2): 189-194.
10. Trippler L, Ali SM, Masoud MO, Mohammed ZH, Amour AK, Suleiman KR, et al. Impact of chemical snail control on intermediate host snail populations for urogenital schistosomiasis elimination in Pemba, Tanzania: findings of a 3-year intervention study. Parasit Vectors. 2024;17(1): 489.
11. Pennance T, Ame SM, Amour AK, Suleiman KR, Cable J, Webster BL. The detection of *Schistosoma bovis* in livestock on Pemba Island, Zanzibar: A preliminary study. Curr Res Parasitol Vector Borne Dis. 2021;1: 100056. Epub 2022/03/15.
12. Pennance T, Ame SM, Amour AK, Suleiman KR, Muhsin MA, Kabole F, et al. Transmission and diversity of *Schistosoma haematobium* and *S. bovis* and their freshwater intermediate snail hosts *Bulinus globosus* and *B. nasutus* in the Zanzibar Archipelago, United Republic of Tanzania. PLoS Negl Trop Dis. 2022;16(7): e0010585. Epub 2022/07/06.
13. Ndum NC, Trippler L, Najim SO, Ali AS, Hattendorf J, Ame SM, et al. One-year impact of behavioural interventions on schistosomiasis-related knowledge, attitude and practices of primary schoolchildren in Pemba, Tanzania. Infect Dis Poverty. 2024;13(1): 84. Epub 20241113.
14. Ndum NC TL, Mohammed UA, Ali MN, Hattendorf J, Ame SM, Kabole F, Utzinger J, Ali SM, Knopp S. Effect of behavioural interventions on schistosomiasis-related knowledge, attitudes and practices of schoolchildren in Pemba, Tanzania: Results of a 3-year study. PLoS Negl Trop Dis. under review.

15. Adriko M, Faust CL, Carruthers LV, Moses A, Tukahebwa EM, Lamberton PHL. Low praziquantel treatment coverage for *Schistosoma mansoni* in Mayuge District, Uganda, due to the absence of treatment opportunities, rather than systematic non-compliance. Trop Med Infect Dis. 2018;3(4). Epub 2018/10/10.
16. Coulibaly JT, Ouattara M, Barda B, Utzinger J, N'Goran EK, Keiser J. A rapid appraisal of factors influencing praziquantel treatment compliance in two communities endemic for schistosomiasis in Côte d'Ivoire. Trop Med Infect Dis. 2018;3(2). Epub 2018/10/03.
17. Dabo A, Bary B, Kouriba B, Sankaré O, Doumbo O. Factors associated with coverage of praziquantel for schistosomiasis control in the community-direct intervention (CDI) approach in Mali (West Africa). Infect Dis Poverty. 2013;2(1): 11.
18. Farrell SH, Truscott JE, Anderson RM. The importance of patient compliance in repeated rounds of mass drug administration (MDA) for the elimination of intestinal helminth transmission. Parasit Vectors. 2017;10(1): 291.
19. Fukushige M, Chase-Topping M, Woolhouse MEJ, Mutapi F. Efficacy of praziquantel has been maintained over four decades (from 1977 to 2018): A systematic review and meta-analysis of factors influence its efficacy. PLoS Negl Trop Dis. 2021;15(3): e0009189. Epub 20210317.
20. Trippler L, Hattendorf J, Ali MN, Najim SO, Khamis KS, Suleiman KR, et al. Evaluation of surveillance-response interventions for *Schistosoma haematobium* elimination on Pemba Island, Tanzania: A 4-years intervention study with repeated cross-sectional surveys. in preparation.
21. Wiegand RE, Fleming FM, Straily A, Montgomery SP, de Vlas SJ, Utzinger J, et al. Urogenital schistosomiasis infection prevalence targets to determine elimination as a public health problem based on microhematuria prevalence in school-age children. PLoS Negl Trop Dis. 2021;15(6): e0009451.
22. Krauth SJ, Greter H, Stete K, Coulibaly JT, Traore SI, Ngandolo BN, et al. All that is blood is not schistosomiasis: experiences with reagent strip testing for urogenital schistosomiasis with special consideration to very-low prevalence settings. Parasit Vectors. 2015;8: 584.
23. C. NN, Trippler L, A. MU, Ali AS, Hattendorf J, Utzinger J, et al. Capacities and needs of health care facilities for schistosomiasis diagnosis and management in elimination settings. Parasit Vectors. 2024.
24. World Health Organization. Schistosomiasis progress report 2001-2011 and strategic plan 2012-2020. World Health Organization, 2013 Report No.: Contract No.
