# Supplementary material for: Adaptive integrated intervention approaches for schistosomiasis elimination in Pemba: A 4-year intervention study and focus on hotspots
Source: PLoS Negl Trop Dis. 2025 Jun 2;19(6):e0013079. doi: 10.1371/journal.pntd.0013079 (PMC12129218; doi:10.1371/journal.pntd.0013079)
Supplement: Attachment — Submitted filename: Minor observations.docx [file pntd.0013079.s006.docx]

Minor observations

- Can author present the data of MDA coverage and compliance to treatment
- Line “8-10mg Niclosamide in 1l – write one litre (it looks like 11)
- Laboratory Examination:- give details of the classification of small and large
- Results:- page 23- line 18- what is large microhaematuria?
- Line 108-118 – “with heavy intensity of infection
